# Pulmonary Arterial Hypertension in Connective Tissue Disorders: The emerging role of screening and early diagnosis. A position paper for Greek Rheumatologists

**DOI:** 10.31138/mjr.30.2.90

**Published:** 2019-06-29

**Authors:** Eftychia Demerouti, Iraklis Tsangaris, Theodoros Dimitroulas, George Giannakoulas, Pelagia Katsimpri, Ioanna Mitrouska, Stylianos Orfanos, Ioannis Skoularigkis, Paraskevi Voulgari, Petros Sfikakis

**Affiliations:** 1 Onassis Cardiac Surgery Center, Pulmonary Hypertension Unit, Athens, Greece,; 2 Attikon University Hospital, Pulmonary Hypertension Clinic, Athens, Greece,; 3 4th Department of Internal Medicine, Hippokration Hospital, Thessaloniki, Greece,; 4 AHEPA University General Hospital, Pulmonary Hypertension Unit, Thessaloniki, Greece,; 5 Rheumatology and Clinical Immunology Unit, “Attikon” University Hospital, Athens, Greece,; 6 Department of Thoracic Medicine, Faculty of Medicine, University of Crete, Crete, Greece,; 7 Department of Cardiology, Larissa University Hospital, Larissa, Greece,; 8 Rheumatology Clinic, Department of Internal Medicine, Medical School, University of Ioannina, Ioannina, Greece,; 9 First Department of Propaedeutic Internal Medicine and Rheumatology Unit, Medical School, National and Kapodistrian University of Athens, Athens, Greece

**Keywords:** Pulmonary arterial hypertension, scleroderma, connective tissue disorders

## Abstract

Pulmonary Arterial Hypertension is regarded as a devastating disease, complicating Connective Tissue Diseases. Although much progress has been achieved in the last 20 years, several unfulfilled needs in diagnosis and management of PAH in these patients may still be identified. After a systematic review of the literature and integrating results from the latest research articles, key clinical issues for the screening and diagnosis of Pulmonary Arterial Hypertension in Connective Tissue Disorder Patients and specifically Scleroderma patients are described in this article, allowing physicians to contribute to early diagnosis of patients with Scleroderma-associated Pulmonary Arterial Hypertension.

## INTRODUCTION

Connective tissue disease (CTD) associated with Pulmonary Arterial Hypertension (PAH) represents the second most common cause of PAH after idiopathic PAH. Amongst CTDs, Systemic Sclerosis (SSc) and SSc spectrum diseases represent the most thoroughly studied diseases associated with PAH. Pulmonary vasculature involvement in CTDs encompasses different pathophysiologic processes; namely, intimal fibrosis, medial hypertrophy, endothelial and smooth muscle cell proliferation, vasculitis, and thromboembolism, all of which may lead to PAH. The latter results in right ventricular dysfunction and death particularly in cases of delayed diagnosis. The therapeutic management has advanced in recent years due to improved understanding in pathophysiology of PAH. However, mortality for SSc patients remains very high. PAH early diagnosis is mandatory, and screening should be performed according to the best evidence-based data we have. The last guidelines for PAH diagnosis^1^ were reported four years ago; however, the 6^th^ World Symposium of Pulmonary Hypertension (6^th^ WSPH) took place in Nice, France in 2018. According to the 6^th^ WSPH Proceedings and recent published data, key clinical issues are necessary to be known for the earlier detection of PAH in patients with CTD. Early diagnosis and management represent a promising strategy to reduce morbidity and mortality in CTDs and specifically SSc patients.

## PULMONARY HYPERTENSION IN SYSTEMIC SCLEROSIS AND SYSTEMIC SCLEROSIS-SPECTRUM DISEASES

Pulmonary Hypertension affects more than 10% of SSc-patients^2^ and may be presented as PH due to Left Heart Disease in the case of Left Ventricular Systolic or Diastolic Dysfunction (Group II), as PH due to Interstitial Lung Disease (Group III), rarely as Chronic Thromboembolic Pulmonary Hypertension (CTEPH) and as PAH (Group I). The prevalence of PAH in SSc patients with diffusing capacity of the lung for carbon monoxide (DLCO) < 60% predicted at time of RHC is 19%.^11^ PAH represents a lethal complication resulting in progressive right ventricular dysfunction, while in Greece it appears to occur in as much as 6–10% in SSc patients. Pulmonary Veno-occlusive disease or Pulmonary Capillary Haemangiomatosis represent rare but increasingly recognized entities, leading to PH. It is of great importance to know that many SSc patients present multifactorial PH with a challenge in its classification in a discrete group because of complex and frequently overlapping clinical features,^3^ as it serves a distinct role in poor outcomes^4^.

Within the last 2 decades, pulmonary fibrosis and PAH have become the leading causes of death in SSc patients.^5^ The estimated 3-year survival rate among patients with PAH associated with SSc is approximately 50%.^6^ Moreover, overall survival in the PHAROS registry^7^ was higher than in other SSc-PAH cohorts. PAH accounted for more than half of the deaths and primarily within the first few years after PAH diagnosis. The increased mortality for SSc-PAH patients highlight the necessity for earlier diagnosis, a process which necessitates prompt screening and right heart catheterization for diagnosis validation.

## PULMONARY ARTERIAL HYPERTENSION HAEMODYNAMIC DEFINITION

The early diagnosis of PAH is mandatory, as early treatment results in better outcome. Diagnosis is based on right heart catheterization (RHC) and precapillary PH detection, which is defined as increased Pulmonary Vascular Resistance (PVR) ≥3WU, mean Pulmonary Arterial Pressure mPAP equal or more than 25mmHg and pulmonary artery wedge pressure (PAWP) ≤15 mmHg, according to the current ESC/ERS Guidelines for PH diagnosis and Treatment.^1^ Recently, in the 6th WSPH, the lower value of 20mmHg mPAP was proposed as new haemodynamic definition of PAH, supported among others by published data indicating that patients with borderline mPAP (mPAP measured between 21 and 24 mmHg) run the risk of disease progression.^8^ In 2013, a single-centre cohort study^9^ of 228 patients with systemic sclerosis was reported; patients with borderline mPAP at diagnosis were more likely to develop overt PAH than patients with mPAP ≤20 mmHg (p<0.001; hazard ratio [HR] 3.7) and five deaths were reported during follow-up, despite specific-PAH drug therapy among patients with borderline mPAP. More recently, a two-centre cohort study^10^ identified 21 patients with SSc and baseline mPAP of 21–24 mmHg, who underwent a second RHC with a median follow-up of 3 years. At baseline, mPAP and PVR were 22±1 mmHg and 2.3±0.8 WU, respectively. At follow-up, mPAP and PVR increased to 25±4 mmHg and 3.2±1.6 WU, respectively. 33% of patients developed overt PH and more specifically, three of them developed PAH.

For many years, the diagnosis of PH was based on an arbitrary value of mPAP ≥25 mmHg, probably because of understandable concerns about overdiagnosis and over-treatment, plus echocardiographic detection limitations.^8^ However, the undertreatment of some patients that carry increased mortality, such as SSc-PAH patients, may result in poor outcomes, and highlights the importance of close monitoring in this population. The lower value of mPAP for PAH diagnosis, when combined with increased PVR, helps to identify a unique, albeit small, population of patients who need close follow-up.^8^ However, the administration of specific PAH drug therapy in this specific population needs further investigation.

## PULMONARY ARTERIAL HYPERTENSION DIAGNOSIS

Pulmonary Arterial Hypertension diagnosis for symptomatic patients is based on a proposed diagnostic algorithm according to ESC/ERS Guidelines for PH diagnosis and treatment.^1^ In symptomatic patients, a transthoracic echocardiogram is performed for PH probability assessment. According to specific recommendations,^1^ in the case of intermediate or high probability, left heart disease, congenital heart disease and lung disease must be excluded. In this step, High-Resolution Spiral Computed Tomography and Pulmonary Functional Test including DLCO is needed. Furthermore, Ventilation-Perfusion Lung Scan must be performed for Chronic Thromboembolic Pulmonary Hypertension exclusion. At this point, attending clinicians must refer the patients to an expert centre for RHC performance and the exclusion of other PH forms for the possible final establishment of PAH diagnosis. Exclusively for SSc patients, the 6^th^ WSPH proceedings^8^ suggest the referral to expert centres early in the diagnostic approach in case intermediate or high probability is assessed in echocardiogram. However, High Resolution Computed Tomography, Spirometry with DLCO estimation and Ventilation-Perfusion Lung Scan can have already been suggested by the Rheumatologist before patient’s referral. The integrated diagnostic approach for all SSc patients is essential since many of them can also be classified in one or more PH groups; notably, the PVOD or PCH must be excluded.

According to ESC/ERS Guidelines,^1^ symptomatic SSc patients must undergo RHC if transthoracic echocardiogram reveals a tricuspid regurgitation velocity more than 2.8 m/sec, but also in the case of a lower tricuspid regurgitation velocity along with other echocardiographic signs. More specifically, if more than two signs of the following three categories are present, RHC must be performed: Category a) Right ventricle/left ventricle basal diameter ratio >1.0 or Flattening of the interventricular septum (left ventricular eccentricity index >1.1 in systole and/or diastole); category b) Right ventricular outflow Doppler acceleration time <105msec or mid-systolic notching or Early diastolic pulmonary regurgitation velocity >2.2 m/sec or PA diameter >25 mm; and category c) Inferior cava diameter >21 mm with decreased inspiratory collapse (<50 % with a sniff or <20 % with quiet inspiration) or right atrial area (end-systole) >18 cm.^2^

The DETECT algorithm^11^ is also recommended for patients with SSc and SSc-spectrum disorders as mixed CTD or other CTDs with prominent scleroderma features as sclerodactyly, nailfold capillary abnormalities and SSc-specific autoantibodies. The screening variables are divided into non-echocardiographic parameters that are often routinely obtained by physicians when dealing with SSc patients, and echocardiographic parameters that are usually available after referring the patient for echocardiography. Step 1 of the algorithm includes the following non-echocardiographic variables: FVC % predicted/DLCO % predicted, current/past telangiectasias, serum anti-centromere antibodies, serum NTproBNP, serum urate, and right axis deviation on ECG. The resulting Step 1 total risk score is used to evaluate whether or not the patient should be referred for echocardiography. Step 2 includes the 2 echocardiographic variables right atrium area and tricuspid regurgitation velocity, as well as the total risk score from Step 1. The patient is assigned risk points for each, and these risk points are added together. The resulting Step 2 total risk score is used to determine if the patient should be referred for right heart catheterization.

## SCREENING FOR PULMONARY ARTERIAL HYPERTENSION DIAGNOSIS IN SCLERODERMA PATIENTS

Survival for SSc-PAH patients remains poor, in part due to the advanced stage at which patients are presented to PAH expert centres. There is considerable lack of robust data estimating the typical duration between the diagnosis of SSc and the diagnosis of PAH. As little or no progress has been made in earlier diagnosis, the experts in the 6^th^ WSPH recommend more aggressive assessment and screening of SSc patients.^12^ For patients with uncorrected DLCO <80% of predicted value, annual screening should be considered. If any of the following screening tests are positive, the experts suggest that the patient must be referred for RHC: a) the DETECT algorithm; b) the 2015 ESC/ERS recommendations for TTE; and c) FVC/DLCO ratio >1.6 (assuming none-to-mild interstitial lung disease) and >2-fold upper limit of normal of NT-proBNP. For patients with uncorrected DLCO ≥80% of predicted, screening may be only considered with TTE.

## KEY CLINICAL ISSUES FOR PULMONARY ARTERIAL HYPERTENSION MANAGEMENT IN CONNECTIVE TISSUE DISORDER PATIENTS: THE TEN COMMANDMENTS FOR THE RHEUMATOLOGIST

- Increased awareness: PAH represents a severe complication in CTD patients, and Rheumatologists should be alerted to its screening and diagnosis.
- Scleroderma patients experience increased morbidity and mortality rates when PAH develops, and, the earlier the diagnosis, the better the outcome.
- Transthoracic echocardiogram is used for detecting PAH according to specific echocardiographic signs, and should be performed by experienced cardiologists. A low estimated PASP in echocardiogram does not exclude PAH diagnosis if other echocardiographic signs are present.
- Screening for asymptomatic patients should be performed annually, by using transthoracic echocardiogram, lung function tests (FVC, DLCO) and NT-proBNP alone, or preferably, in combination.
- In case of an uncorrected DLCO lower than 80%, or increased NT-proBNP or FVC/DLCO>1.6, or transthoracic echocardiogram with intermediate or high probability for PAH, referral to expert centres is necessary.
- Connective Tissue Disorder Patients may develop pulmonary hypertension due to different pathophysiologic mechanisms upon diagnosis or over time. Reclassification of patients over time is not uncommon.
- High Resolution Computed Tomography and Ventilation/Perfusion Lung Scan are significant parts of the initial diagnostic work up.
- Right Heart Catheterization is required for PAH diagnosis.
- PAH-specific drug therapy should not be initiated without baseline hemodynamic parameters, as they are necessary not only to establish PAH diagnosis, but also for the patient’s risk stratification and their prompt follow-up.
- An estimated mean Pulmonary Arterial Pressure by Right Heart Catheterization between 21 and 25mmHg implies a closer patient’s follow-up. Given the lack of studies, the question of PAH-specific drug initiation in this category of patients remains open and should be decided on an individual basis.

## CONCLUSION

The Rheumatologist should be alerted to the possibility of PAH presence in CTD patients. Prompt screening is mandatory for the diagnosis in earlier stages in the course of PAH, but the overall therapeutic management is better achieved after referral to expert Pulmonary Hypertension Centres with a multidisciplinary team including rheumatologists, pulmonologists and cardiologists. Effective collaboration of the referring Rheumatologist and the expert PH centre is of paramount importance for the management of such patients.
